# Statistical Characterization and Modeling of Indoor RF-EMF Down-Link Exposure

**DOI:** 10.3390/s23073583

**Published:** 2023-03-29

**Authors:** Biruk Ashenafi Mulugeta, Shanshan Wang, Wassim Ben Chikha, Jiang Liu, Christophe Roblin, Joe Wiart

**Affiliations:** Chaire C2M, LTCI, Telecom Paris, Institut Polytechnique de Paris, 91120 Palaiseau, France; biruk.mulugeta@telecom-paris.fr (B.A.M.); wassim.benchikha@telecom-paris.fr (W.B.C.); jiang.liu@telecom-paris.fr (J.L.); christophe.roblin@telecom-paris.fr (C.R.); joe.wiart@telecom-paris.fr (J.W.)

**Keywords:** indoor, exposure, RF-EMF, measurement, Kolmogorov–Smirnov, statistics, down-link, Gaussian

## Abstract

With the increasing use of wireless communication systems, assessment of exposure to radio-frequency electromagnetic field (RF-EMF) has now become very important due to the rise of public risk perception. Since people spend more than 70% of their daily time in indoor environments, including home, office, and car, the efforts devoted to indoor RF-EMF exposure assessment has also increased. However, assessment of indoor exposure to RF-EMF using a deterministic approach is challenging and time consuming task as it is affected by uncertainties due to the complexity of the indoor environment and furniture structure, existence of multiple reflection, refraction, diffraction and scattering, temporal variability of exposure, and existence of many obstructions with unknown dielectric properties. Moreover, it is also affected by the existence of uncontrolled factors that can influence the indoor RF-EMF exposure such as the constant movement of people and random movement of furniture and doors as people are working in the building. In this study, a statistical approach is utilized to characterize and model the total indoor RF-EMF down-link (DL) exposure from all cellular bands on each floor over the length of a wing since the significance of distance is very low between any two points on each floor in a wing and the variation of RF-EMF DL exposure is mainly influenced by the local indoor environment. Measurements were conducted in three buildings that are located within a few hundred meters vicinity of two base station sites supporting several cellular technologies (2G, 3G, 4G, and 5G). We apply the one-sample Kolmogorov–Smirnov test on the measurement data, and we prove that the indoor RF-EMF DL exposure on each floor over the length of a wing is a random process governed by a Gaussian distribution. We validate this proposition using leave-one-out cross validation technique. Consequently, we conclude that the indoor RF-EMF DL exposure on each floor over the length of a wing can be modeled by a Gaussian distribution and, therefore, can be characterized by the mean and the standard deviation parameters.

## 1. Introduction

Since the last decade, radio technologies have undergone a rapid evolution to fulfill the growing needs to connect virtually everyone and everything together, including machines, devices, and objects. With this increasing use of wireless communication systems and connected objects, the question of the health impact of radio-frequency (RF) waves and the public risk perception has arisen. As people spend more than 70% of their daily time in indoor environments, the efforts devoted to indoor RF-EMF exposure assessment has also been increased [1,2]. Indeed, despite the increasing usage and weak exposure, the concern related to electro-magnetic field (EMF) exposure is important [3,4]. International guidelines, such as ICNIRP [5] and IEEE C95.1 [6], have been established to avoid over exposure that may induce adverse health effects.

Since the power level attenuation can reach up to 20 dB when electromagnetic (EM) wave propagates from outdoor to indoor [7], indoor antennas are sometimes installed in some indoor environments to enhance the indoor coverage. This reduces the user equipment (UE) power consumption as the transmitted power from the UE will be reduced by the up-link power control scheme.

Indoor environments are composed of furniture, walls, floors, windows, doors, and partitions of different materials. These objects determine the way in which electromagnetic waves propagate along specific paths. Because of this, EM waves suffer from multiple attenuation, reflection, refraction, diffraction, and scattering which make the deterministic assessment of indoor RF-EMF exposure challenging and time-consuming task.

Several studies aimed to estimate the indoor RF-EMF exposure in the frequency range of 10 MHz to 6 GHz [8,9,10,11,12,13,14]. The most accurate method to estimate the indoor RF-EMF exposure is by directly solving the Maxwell’s equations using full wave deterministic technique, but it is not suitable for large indoor environment as it requires detailed information about the environment, which induces memory load and high computational cost [8,9,10]. For large indoor environment, ray tracing and ray launching deterministic techniques offer a good approximation with relatively lower computational cost [11,12,13]. However, it is very difficult to utilize these deterministic approaches for the assessment of indoor RF-EMF exposure if the indoor environment includes many moving objects and the dielectric properties and geometries of all objects are unknown. In this sense, statistical approach provides a good approximation. In [15], it has been proved that the distribution of the received signal strength from a single base station on some arbitrary point inside an assembly hall turns out to be Gaussian. In [16], the received power at 60 GHz for fifth generation (5G) millimeter-wave (mmWave) wireless communication systems in an indoor environment is subject to the normal distribution. The authors of [15] and [16] studied the law that governs the received signal strength distribution for indoor mobile users positioning system and propagation characteristic of mmWave signal in indoor radio channels based on the method of shooting and bouncing ray tracing/image, respectively.

In this paper, a statistical approach is utilized to characterize and model indoor RF-EMF down-link (DL) exposure. Measurements were conducted in the corridors and some offices of three buildings. The access to these buildings, which are located in Les Clayes sous Bois, has been authorized by ATOS. The buildings are located within a few hundred meters vicinity of two base station sites with several cellular antennas (2G, 3G, 4G, and 5G) from four operators. We aim to characterize the total indoor RF-EMF DL exposure from all cellular bands on each floor over the length of a wing where the significance of distance is very low between any two points and the variation of RF-EMF DL exposure is mainly caused by the local indoor environment. First, the contribution of each band to the total exposure is investigated to identify the base stations that induce the incident field. Next, the one-sample Kolmogorov–Smirnov (K-S) test is applied on the measurement data to check if the indoor RF-EMF DL exposure on each floor over the length of a wing is a random process governed by a Gaussian distribution. Afterwards, the model is cross-checked using the leave-one cross validation technique to check if the distribution is still governed by the same statistical law. Finally, the influence of floor level on the mean indoor RF-EMF DL exposure is investigated.

The remainder of this paper is organized as follows: we describe the material and method in Section 2. In Section 3, we analyze the results. Finally, we conclude this paper with Section 4.

## 2. Material and Method

In this study, we used both frequency-selective and broadband measurement systems. The frequency-selective measurement system is dedicated to record the time variation linked to the traffic change over time which is used for the normalization of the spatial measurements. Whereas, the broadband measurement system is dedicated for the spatial measurements carried out in different buildings and wings.

### 2.1. Frequency-Selective Measurement System

**Figure 1:**
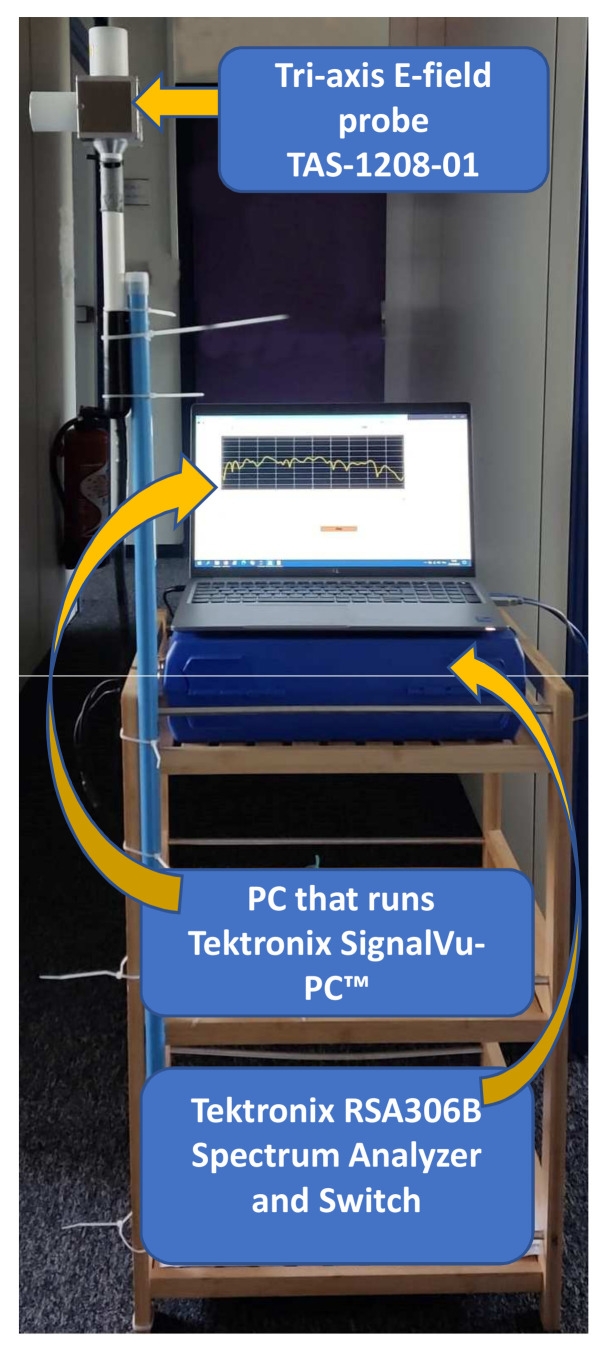
Real-time spectrum analyzer EMF measurement system.

The frequency-selective measurement system used for continuous temporal measurements in this study consists of a real-time spectrum analyzer, i.e., Tektronix RSA306B, switch, Arduino-based hardware, tri-axis electric field (E-field) probe, a PC that runs Tektronix SignalVu-PC™ RF signal analysis software and a graphical user interface (GUI) to control the measurements as shown in Figure 1.

The tri-axis E-field probe, which is commercialized by Microwave Vision Group (MVG) as TAS-1208-01 antenna, is used to conduct measurement of RF-EMF exposure on the three orthogonal polarizations (X, Y, and Z). Our frequency-selective measurement system allows measurements from 9 kHz to 6.2 GHz. The RF switch connects the spectrum analyzer and the tri-axis E-field probe to conduct measurements on the three orthogonal polarizations.

We have one measurement of the selected band at a time if we use only the Tektronix SignalVu-PC™ RF signal analysis software interface. Therefore, we developed a GUI that is synchronized with the SignalVu-PC software to control all measurement parameters and to fetch real-time measurement values continuously. Calibration was performed in the laboratory and an anechoic chamber to maintain the measurement system’s accuracy.

The frequency bands, that are under analysis, are the ones used by the network providers in France as given by ANFR [17]. The resolution bandwidth (RBW) is set to 250 kHz for for all bands. For each measurement location, the frequency-selective measurement system recursively measures the E-field induced by 27 frequency bands (cellular bands used by all network providers in France) on a single axis before switching to the other axes for 20 records. The total E-field is, thus, the root-mean square of the E-field measured on each axis.

Given the fact that we set the number of records to 20, we need a time duration of about 15 min to record and compute the E-field at a given measurement location, which is too long compared to the Narda NBM-550 broadband measurement system. In fact, the Narda NBM-550 measures 100 records per minute. Therefore, we decided to use broadband system for spatial measurements throughout the three buildings. The broadband measurement system is described in Section 2.2.

### 2.2. Broadband Measurement System

**Figure 2:**
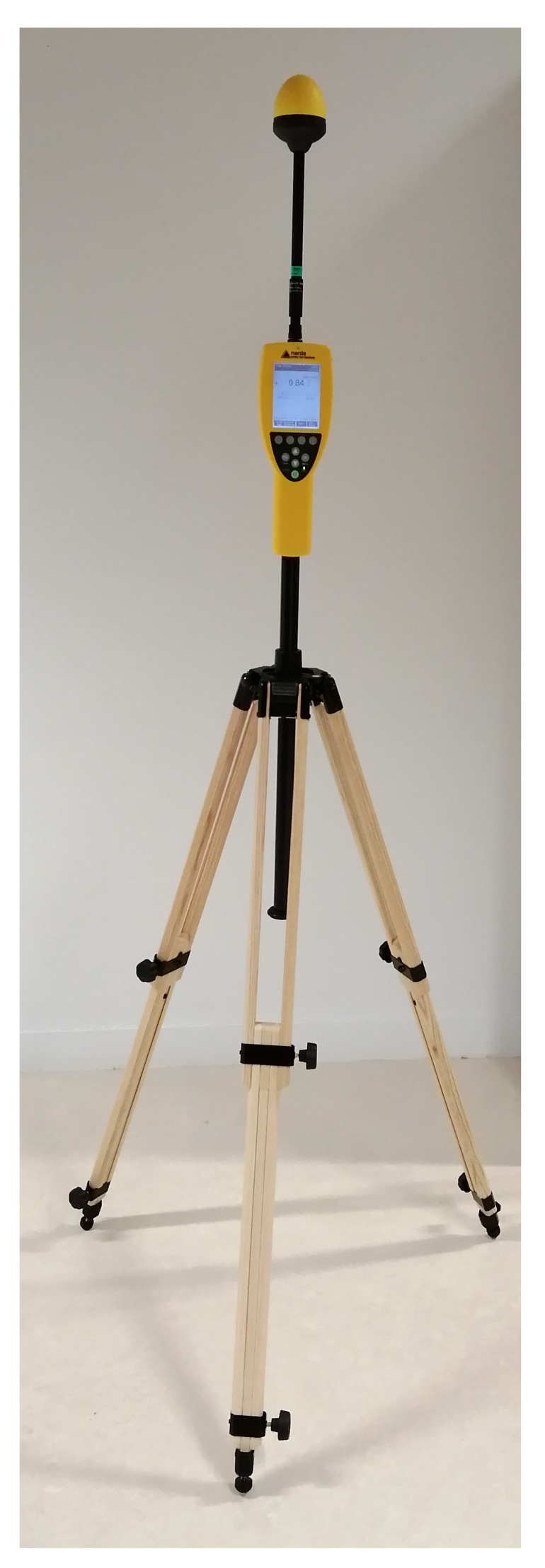
Narda NBM-550 broadband measurement system.

The broadband measurement system is commercialized by Narda as NBM-550 broadband field meter with isotropic EF0691 probe as shown in Figure 2. The probe detects electric fields from 100 kHz to 6 GHz.

At a given measurement location, 100 broadband measurements were recorded in one minute as the broadband measurement system records the RF-EMF exposure every 0.6 s.

### 2.3. Measurement Procedure Description

**Figure 3:**
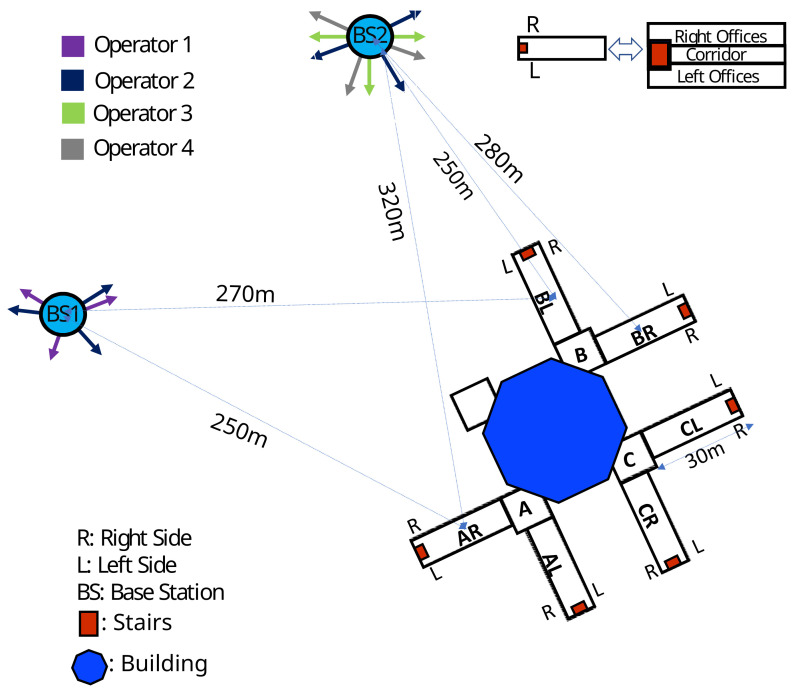
Building plans with respect to the base stations.

Broadband measurements were conducted at two different probe heights (1.2 m and 1.7 m) in the corridors and some offices of three buildings (A, B, and C) which are shown in Figure 3. In total, 1080 spatial measurements were conducted in the corridors of all buildings (30 measurement locations × 2 heights × 3 floors × 2 wings × 3 buildings) with 1 m separation distance to investigate the statistical law governing the exposure distribution in the indoor environment as it is necessary to estimate the exposure level as a function of the spatial distribution of the measurements [18]. Moreover, 96 spatial measurements (16 measurement points × 2 heights × 1 floors × 1 wings × 3 buildings) were also conducted in the offices on the second floor of one wing of each building (A2R, B2L, and C2L). The ground, first and second floors are labeled 0, 1, and 2, respectively, in between the labels of each wing (for example, the first floor of AR is labeled A1R). The internal walls of buildings A, B, and C are mostly metal, plaster, and metal, respectively. The second floor of BL and CL (B2L and C2L) wings were empty and there were a random movement of people in other part of the buildings while we were conducting the measurements.

Measurement results exhibit variations in both spatial and time domains due to radio channel and traffic variations [19]. Radio channel quality varies by the distance to the base station, random environmental variation, and interference variation. Whereas, traffic pattern varies by user demand and server load. It is, therefore, important to have an appropriate measurement strategy that takes such variations into account and reduces the dynamics of mobile data traffic from the spatial measurements. While conducting the broadband spatial measurements, frequency-selective temporal measurement was launched at a stationary position in an office of one of the buildings, which has a clear view of the base stations, as a reference measurement to monitor the time variations linked with the traffic change over time. Since the spatial measurements depend on both location and temporal traffic, the temporal measurement is used for normalization of the spatial measurements.

### 2.4. Field Strength Normalization

In the use of wireless communication, the traffic has an influence on the field emitted by the base station. Since the measurement time of each measurement point is different, the traffic is also different. In this study, we are interested in analyzing the spatial variation of broadband measurements. Therefore, the traffic variation has to be taken into consideration and the measurements should be transformed into their equivalent form at the same reference time. Otherwise, it is difficult to identify the cause of measurement variations as the measurement value is influenced by both spatial and temporal variations.

The time for the first spatial measurement point, denoted by $t_{i=1}$, was chosen as a reference time. The reference electric field ($E_{\mathrm{ref}}$) is, then, extracted from the temporal measurement for each spatial measurement point based on the time of measurement. After extracting the reference temporal measurement ($E_{\mathrm{ref}}\left(t_{i}\right)$) for each measurement point, the field measured at a given measurement point “*i*” has to be weighted by a correction factor of $\frac{E_{\mathrm{ref}}\left(t_{i=1}\right)}{E_{\mathrm{ref}}\left(t_{i}\right)}$ in order to transform in to its equivalent form at “$t_{i=1}$” ($E_{t_{i}\_\mathrm{eq}}\left(t_{i=1}\right)$) [20]. The whole spatial measurements are transformed to their equivalent form at the time of the first measurement point ($E_{i\_\mathrm{eq}}\left(t_{i=1}\right)$) based on Equation (1).

(1) $$E_{t_{i}\_\mathrm{eq}}\left(t_{i=1}\right)=E\left(t_{i}\right)*\frac{E_{\mathrm{ref}}\left(t_{i=1}\right)}{E_{\mathrm{ref}}\left(t_{i}\right)}$$

### 2.5. K-S Test for RF-EMF DL Exposure Statistical Modeling

In this subsection, we describe how to use a statistical approach to characterize indoor RF-EMF DL exposure. The null hypothesis is defined as “the indoor RF-EMF DL exposure is a random process governed by Gaussian distribution on each floor over the length of a wing when the indoor environment is located within a few hundred meters vicinity of base station sites”. We use the measurement data to test and validate a null hypothesis using the one-sample K-S test. K-S test is a non-parametric test that can be used to compare a collection of samples with a reference probability distribution.

The K-S test statistic quantifies the distance between the empirical cumulative distribution function (CDF) of the sample and the CDF of the reference distribution. In contrast, *p*-values are often interpreted as the risk of rejecting the null hypothesis of the test when the null hypothesis is actually true [21]. This probability reflects the measure of evidence against the null hypothesis. Small *p*-values (less than the significance level which is 0.05 in our case) correspond to strong evidence against the null hypothesis. If the *p*-value is greater than the significance level, then we fail to reject the null hypothesis.

## 3. Result and Discussion

### 3.1. Time Variation of Electric Field

As explained in Section 2.3, we use the frequency-selective reference temporal measurement to record the time variation linked to the traffic change over time. We use this temporal measurement to normalize the spatial measurements as shown in Equation (1).

The electric field of each band ($E_{i}\left(t\right))$ is measured on each axis ($Ex_{i}\left(t\right)$, $Ey_{i}\left(t\right)$ and $Ez_{i}\left(t\right))$ and the total electric field is computed using Equation (2).

(2) $$E_{\mathrm{total}}\left(t\right)=\sqrt{\sum_{i\in f}E_{i}^{2}\left(t\right)}$$

where, f={700MHz,800MHz,900MHz,1800MHz,2100MHz,2600MHz,3500MHz} and $E_{i}\left(t\right)=\sqrt{{\mathrm{Ex}}_{i}^{2}\left(t\right)+{\mathrm{Ey}}_{i}^{2}\left(t\right)+{\mathrm{Ez}}_{i}^{2}\left(t\right)}$

**Figure 4:**
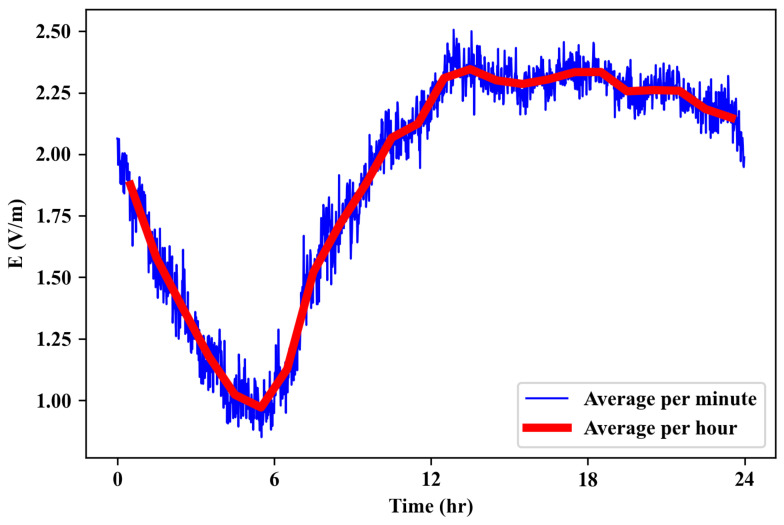
Time variation of electric field in 24 h.

In Figure 4, we plot the temporal variation of the total electric field over 24 h averaged per minute and per hour. As seen from this result, the RF-EMF DL exposure level decreases at night and then increases during the daytime due to the significant increase in cellular usage.

### 3.2. Frequency-Selective Measurement

The nearby base stations, which are located within the vicinity of the buildings, support several cellular antennas on the 700 MHz, 800 MHz, 900 MHz, 1800 MHz, 2100 MHz, 2600 MHz, and 3500 MHz frequency bands for mobile communications. Of the new generation technologies, 5G operates on multiple bands (700 MHz, 2100 MHz, and 3500 MHz) and LTE operates on all bands except 900 MHz and 3500 MHz bands [17]. In contrast, 2G and 3G operate on 900 MHz band as shown in Table 1.

**Figure 5:**
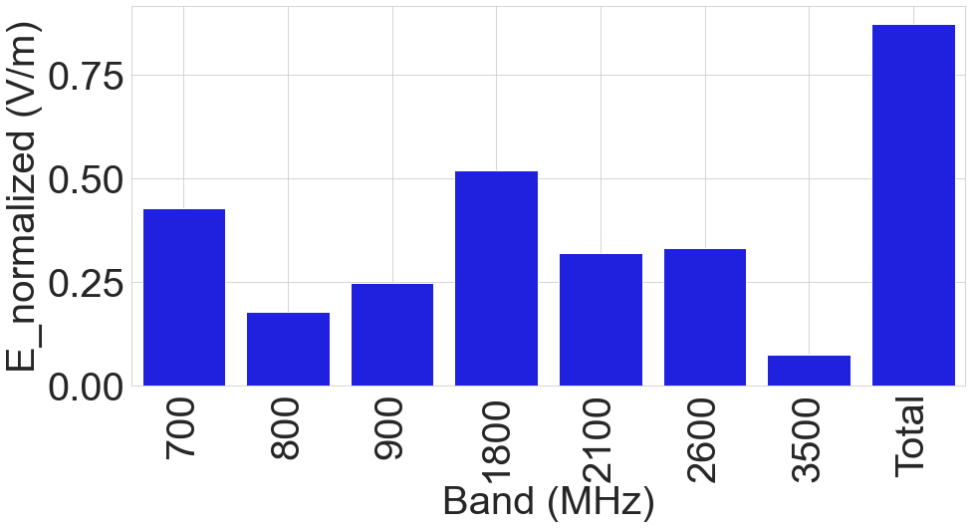
Mean contribution of each band on total **E** field.

The result of the frequency-selective measurements performed inside the building is shown in Figure 5 where the total electric field is computed using Equation (2). It shows significant contributions from all cellular bands listed in Table 1, which is coherent with nearby base station antenna information, according to Cartoradio [17].

### 3.3. Broadband Measurement

**Figure 6:**
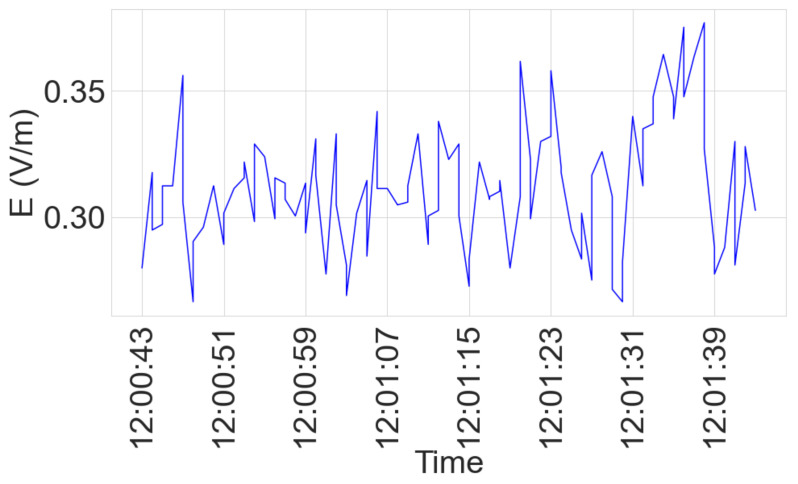
Broadband measurement variability over one minute.

Figure 6 shows the variability of the broadband measurement over one minute at a given measurement point.

The analysis in the subsequent sections takes the mean of measurements performed in one minute for a given measurement point. Therefore, uncertainties are introduced in our measurement analysis due to the variation over one minute.

### 3.4. Different Probe Height Measurements

The mean and standard deviation of the two measurement heights (1.2 m and 1.7 m) on all wings of the buildings are listed in Table 2, Table 3 and Table 4 for building A, B, and C, respectively.

In Table 5, we compute the Pearson correlation coefficients between the statistical parameters (i.e., mean, median, and standard deviation) of the two probe heights according to the values presented in the Table 2, Table 3 and Table 4 for the buildings A, B, and C, respectively. As seen from these results, the Pearson correlation coefficients on each building are close to 1. Hence, the measurements at the two probe heights are highly correlated in terms of mean, median, and standard deviation. Consequently, we can consider that the exposure level for these two heights are similar. In this case, we merge the measurements at the two probe heights for the subsequent measurement analysis.

### 3.5. Statistical Analysis of Measurements

**Figure 7:**
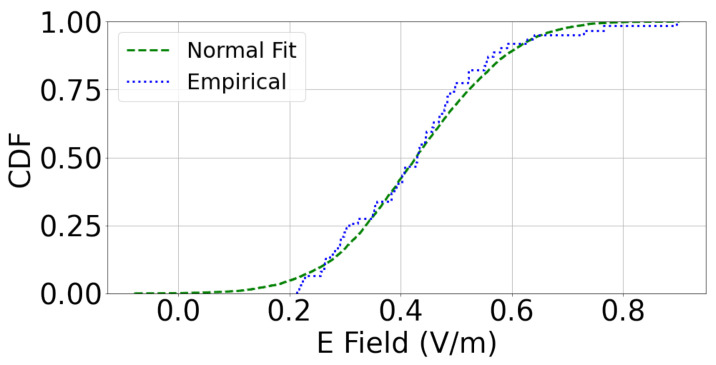
The CDFs of normal distribution and the empirically observed distribution of A1L.

In order to characterize the indoor RF-EMF DL exposure in different buildings’ corridors, we apply the one-sample K-S test, which is described in Section 2.5. Under the null hypothesis, we find out that the *p*-values on all floors of the three buildings are greater than 0.05 with lower test statistic as shown in Table 6. For example, Figure 7 shows a normal distribution that fits the empirically observed distribution of A1L wing with 0.08 K-S test statistic, which is the length of the largest vertical line we could draw between the two CDFs. This proves the null hypothesis by not rejecting it with 0.05 significance level and 95% confidence level, which shows the percentage of times we expect to come close to the same estimate if we run our experiment again. Moreover, the Pearson correlation coefficients between the mean and the median are 0.995, 0.985 and 0.995 for the three buildings A, B and C, respectively. Therefore, we can conclude that the indoor RF-EMF DL exposure on each floor over the length of a wing can be modeled as a random process governed by a Gaussian distribution. Moreover, the normality of the distribution was not affected by the presence (Status is “Occupied”) and absence of people (Status is “Empty”) as shown in Table 6.

Accordingly, we can model the indoor RF-EMF DL exposure on each floor over the length of a wing using a Gaussian distribution and characterize it by only the mean and standard deviation parameters. To confirm this result, we perform the leave-one-out cross-validation technique in the next subsection.

### 3.6. Leave-One-Out Cross-Validation

In this subsection, we cross-check the validity of our model using the leave-one-out cross-validation technique. It is used to check the robustness of normal distribution of the wings by leaving one measurement point out at a time in an iterative way for all measurement points of wings on each floor. Then, we statistically prove that indoor RF-EMF DL exposure on each floor over the length of a wing is governed by Gaussian distribution with a probability of 0.973 and 1 for the corridors and the offices, respectively.

**Figure 8:**
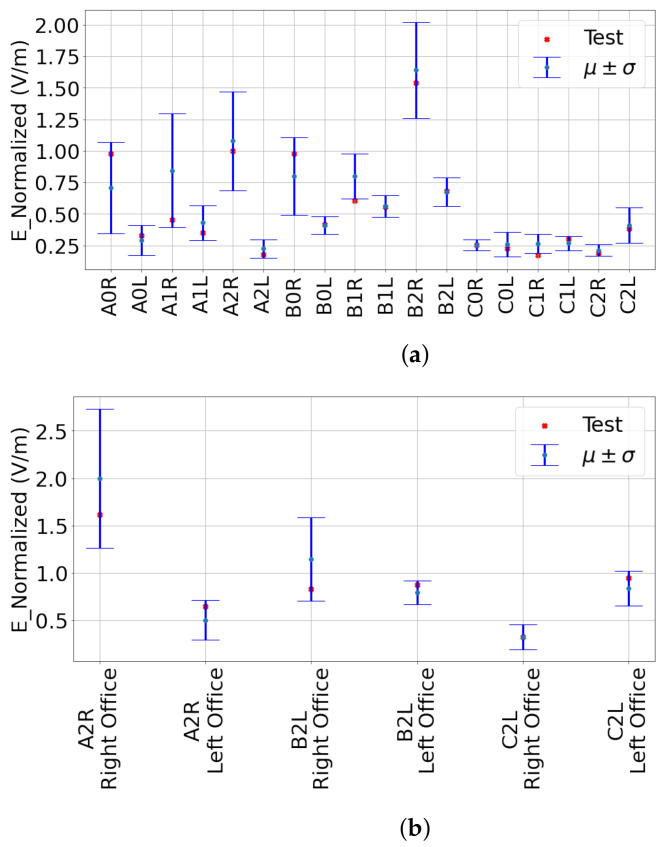
Validation of test point within one standard error from the mean in the wings of all buildings. (a) Corridors; (b) Offices.

In Figure 8, uncertainties on error bar plots are shown to indicate where randomly chosen test data from N measurement data points will fall within one standard deviation from the mean of the rest (N-1) measurement data points.

### 3.7. The Influence of Floor Level on the Mean Indoor RF-EMF DL Exposure

The indoor RF-EMF DL exposure is influenced by the floor level of a building [22,23]. The floor level of a building determines the beam down tilt angle which in turn determines the antenna gain. The antenna gain reduces with an increase in the angle in going up and down beyond the peak of the main beam. The beam down tilt angle depends on the ratio of height difference and distance difference, between antenna and receiver [24,25]. We use the AR wing to check the floor level influence on the indoor RF-EMF DL exposure since it has a clear view of the two base stations. In the AR wing, as shown in Table 7, the ratios of the mean exposure level on the second floor to the corresponding level on the first floor and the mean exposure level on the first floor to the corresponding level on the ground floor are 1.29 (2.2 dB) and 1.16 (1.3 dB), respectively. This implies that the indoor RF-EMF DL exposure increases with an increase in floor level. The measurement antenna height increases by three meters with the floor level as the height of each floor is three meters. The beam angle difference between each floor level will decrease when the distance between the measurement point and the base station increases. Moreover, the maximum RF-EMF DL exposure levels in all floors are well below the ICNIRP limits.

## 4. Conclusions

This paper analyzes the indoor RF-EMF DL exposure with outdoor cellular antennas located at more than 200 m from the buildings. In the three buildings, 1176 measurements have been performed with a broadband probe at both corridors and offices on different floors. With the base station antenna far away, the exposure is well below 1% of the ICNIRP reference levels as expected.

A statistical approach has been implemented to characterize and model the indoor RF-EMF DL exposure. The measurement data were analyzed and the *p*-values of the one-sample K-S test are above 0.05. Therefore, it has been statistically proved that the indoor RF-EMF DL exposure on each floor over the length of a wing can be modeled by a Gaussian distribution when the size of the building is small compared to the distance to the base station antennas. In such case, the mean and the standard deviation characterize the RF-EMF DL exposure distribution in the indoor environment.

Finally, the result of this work can be used as a step-stone to install a global indoor RF-EMF DL exposure monitoring system in ATOS via the implementation of measurements carried out by RF sensors distributed in the buildings.

## Figures and Tables

**Table 1:** Frequency bands utilized by different mobile communication technologies.

Band	Technology
700	4G, 5G
800	4G
900	2G, 3G
1800	4G
2100	3G, 4G, 5G
2600	4G
3500	5G

**Table 2:** Mean, median, and standard deviation of two height measurements for building A.

Wing	Type	Height	Mean	Median	Std
		(m)	(V/m)	(V/m)	(V/m)
A0R	Corridor	1.2	0.753	0.776	0.385
		1.7	0.680	0.687	0.326
A0L	Corridor	1.2	0.313	0.277	0.126
		1.7	0.275	0.238	0.102
A1R	Corridor	1.2	0.922	0.764	0.495
		1.7	0.747	0.632	0.375
A1L	Corridor	1.2	0.417	0.429	0.124
		1.7	0.439	0.429	0.148
A2R	Corridor	1.2	1.104	1.100	0.398
		1.7	1.051	1.031	0.373
	R Office	1.2	1.956	2.028	0.674
		1.7	1.947	2.001	0.724
	L Office	1.2	0.526	0.565	0.197
		1.7	0.515	0.544	0.209
A2L	Corridor	1.2	0.202	0.193	0.052
		1.7	0.246	0.236	0.081

**Table 3:** Mean, median, and standard deviation of two height measurements for building B.

Wing	Type	Height	Mean	Median	Std
		(m)	(V/m)	(V/m)	(V/m)
B0R	Corridor	1.2	0.843	0.715	0.346
		1.7	0.766	0.753	0.249
B0L	Corridor	1.2	0.420	0.431	0.056
		1.7	0.401	0.417	0.081
B1R	Corridor	1.2	0.866	0.842	0.174
		1.7	0.722	0.707	0.156
B1L	Corridor	1.2	0.591	0.579	0.088
		1.7	0.532	0.526	0.073
B2R	Corridor	1.2	1.812	1.729	0.419
		1.7	1.461	1.442	0.214
B2L	Corridor	1.2	0.691	0.705	0.106
		1.7	0.660	0.670	0.112
	R Office	1.2	1.155	0.930	0.495
		1.7	1.056	0.917	0.342
	L Office	1.2	0.804	0.827	0.132
		1.7	0.797	0.768	0.105

**Table 4:** Mean, median, and standard deviation of two height measurements for building C.

Wing	Type	Height	Mean	Median	Std
		(m)	(V/m)	(V/m)	(V/m)
C0R	Corridor	1.2	0.246	0.252	0.035
		1.7	0.26	0.244	0.049
C0L	Corridor	1.2	0.264	0.246	0.098
		1.7	0.254	0.231	0.093
C1R	Corridor	1.2	0.254	0.249	0.066
		1.7	0.268	0.234	0.085
C1L	Corridor	1.2	0.268	0.273	0.055
		1.7	0.271	0.266	0.059
C2R	Corridor	1.2	0.199	0.193	0.040
		1.7	0.224	0.217	0.048
C2L	Corridor	1.2	0.455	0.491	0.152
		1.7	0.365	0.348	0.106
	R Office	1.2	0.335	0.263	0.14
		1.7	0.312	0.277	0.104
	L Office	1.2	0.790	0.781	0.151
		1.7	0.911	0.926	0.186

**Table 5:** Pearson correlation coefficients between the measurements performed at the two probe heights.

Building	Correlation Coefficient		
	Mean	Median	Std
A	0.993	0.996	0.966
B	0.990	0.985	0.956
C	0.974	0.982	0.836

**Table 6:** K-S Test checking the normality of exposure on all wings of building A, B, and C.

Wing	Type	Status	Test Statistic	*p*-Value
A0R	Corridor	Occupied	0.118	0.308
A0L	Corridor	Occupied	0.133	0.187
A1R	Corridor	Occupied	0.147	0.086
A1L	Corridor	Occupied	0.08	0.795
A2R	Corridor	Occupied	0.089	0.599
	R Office	Occupied	0.144	0.848
	L Office	Occupied	0.166	0.776
A2L	Corridor	Occupied	0.114	0.363
B0R	Corridor	Occupied	0.131	0.287
B0L	Corridor	Occupied	0.139	0.244
B1R	Corridor	Occupied	0.12	0.365
B1L	Corridor	Occupied	0.085	0.814
B2R	Corridor	Occupied	0.173	0.062
B2L	Corridor	Empty	0.138	0.25
	R Office	Empty	0.248	0.235
	L Office	Empty	0.114	0.932
C0R	Corridor	Occupied	0.091	0.991
C0L	Corridor	Occupied	0.173	0.078
C1R	Corridor	Occupied	0.104	0.507
C1L	Corridor	Occupied	0.089	0.656
C2R	Corridor	Occupied	0.106	0.498
C2L	Corridor	Empty	0.162	0.056
	R Office	Empty	0.251	0.172
	L Office	Empty	0.130	0.916

**Table 7:** Mean, maximum, and standard deviation of measurements in A, B, and C wings.

Wing	Wing-Floor	Type	μ	σ	Max	σ/μ	Wing-Mean
			(V/m)	(V/m)	(V/m)		(V/m)
AR	A0R	Corridor	0.716	0.361	1.540	0.5	0.88
	A1R	Corridor	0.834	0.451	2.309	0.5	
	A2R	Corridor	1.077	0.389	1.956	0.4	
		R Office	1.951	0.722	3.448	0.4	
		L Office	0.521	0.211	0.818	0.4	
AL	A0L	Corridor	0.294	0.117	0.662	0.4	0.32
	A1L	Corridor	0.428	0.138	0.897	0.3	
	A2L	Corridor	0.224	0.072	0.494	0.3	
BR	B0R	Corridor	0.804	0.307	1.796	0.4	1.08
	B1R	Corridor	0.793	0.182	1.288	0.2	
	B2R	Corridor	1.636	0.38	2.906	0.2	
BL	B0L	Corridor	0.411	0.071	0.526	0.2	0.55
	B1L	Corridor	0.562	0.087	0.841	0.2	
	B2L	Corridor	0.675	0.111	0.863	0.2	
		R Office	1.105	0.442	2.334	0.4	
		L Office	0.801	0.122	1.042	0.2	
CR	C0R	Corridor	0.253	0.045	0.348	0.2	0.25
	C1R	Corridor	0.261	0.077	0.464	0.3	
	C2R	Corridor	0.212	0.046	0.317	0.2	
CL	C0L	Corridor	0.259	0.096	0.610	0.4	0.31
	C1L	Corridor	0.270	0.057	0.422	0.2	
	C2L	Corridor	0.410	0.139	0.859	0.3	
		R Office	0.324	0.127	0.652	0.4	
		L Office	0.851	0.186	1.232	0.2	

## Data Availability

The data presented in this study are available on request from the corresponding author.

## Abbreviations

The following abbreviations are used in this manuscript:

RF	Radio Frequency
EMF	Electro-Magnetic Field
DL	Down-Link
ICNIRP	International Commission on Non-Ionizing Radiation Protection
IEEE	Institute of Electrical and Electronics Engineers
UE	User Equipment
K-S	Kolmogorov–Smirnov
GUI	Graphical User Interface
MVG	Microwave Vision Group
ANFR	L’Agence Nationale des FRéquences
RBW	Resolution Band Width
CDFs	Cumulative Distribution Functions
